# Pharmacological Treatment of Advanced, Persistent or Metastatic Endometrial Cancer: State of the Art and Perspectives of Clinical Research for the Special Issue “Diagnosis and Management of Endometrial Cancer”

**DOI:** 10.3390/cancers13246155

**Published:** 2021-12-07

**Authors:** Angiolo Gadducci, Stefania Cosio

**Affiliations:** Department of Clinical and Experimental Medicine, Division of Gynecology and Obstetrics, University of Pisa, 56127 Pisa, Italy; stefania.cosio@gmail.com

**Keywords:** endometrial carcinoma, chemotherapy, hormonal therapy, biological agents, immune checkpoint inhibitors

## Abstract

**Simple Summary:**

Patients with metastatic or recurrent endometrial cancer (EC) not suitable for surgery and/or radiotherapy are candidates for pharmacological treatment with unsatisfactory clinical outcomes. The combination of carboplatin + paclitaxel is the standard first-line chemotherapy, whereas hormonal therapy is sometimes used in selected patients with slow-growing steroid receptor-positive EC. The combination of endocrine therapy with mTOR inhibitors or cyclin-dependent kinase 4/6 inhibitors is currently under evaluation. Immune checkpoint inhibitors, and especially pembrolizumab and dostarlimab, have achieved an objective response in 27–47% of highly pretreated patients with microsatellite instability-high/mismatch repair (MMR)-deficient EC.

**Abstract:**

Patients with metastatic or recurrent endometrial cancer (EC) not suitable for surgery and/or radiotherapy are candidates for pharmacological treatment frequently with unsatisfactory clinical outcomes. The purpose of this paper was to review the results obtained with chemotherapy, hormonal therapy, biological agents and immune checkpoint inhibitors in this clinical setting. The combination of carboplatin (CBDCA) + paclitaxel (PTX) is the standard first-line chemotherapy capable of achieving objective response rates (ORRs) of 43–62%, a median progression-free survival (PFS) of 5.3–15 months and a median overall survival (OS) of 13.2–37.0 months, respectively, whereas hormonal therapy is sometimes used in selected patients with slow-growing steroid receptor-positive EC. The combination of endocrine therapy with m-TOR inhibitors or cyclin-dependent kinase 4/6 inhibitors is currently under evaluation. Disappointing ORRs have been associated with epidermal growth factor receptor (EGFR) inhibitors, HER-2 inhibitors and multi-tyrosine kinase inhibitors used as single agents, and clinical trials evaluating the addition of bevacizumab to CBDCA + PTX have reported conflicting results. Immune checkpoint inhibitors, and especially pembrolizumab and dostarlimab, have achieved an objective response in 27–47% of highly pretreated patients with microsatellite instability-high (MSI-H)/mismatch repair (MMR)-deficient (-d) EC. In a recent study, the combination of lenvatinib + pembrolizumab produced a 24-week response rate of 38% in patients with highly pretreated EC, ranging from 64% in patients with MSI-H/MMR-d to 36% in those with microsatellite stable/MMR-proficient tumors. Four trials are currently investigating the addition of immune checkpoint inhibitors to PTX + CBDCA in primary advanced or recurrent EC, and two trials are comparing pembrolizumab + lenvatinib versus either CBDCA + PTX as a first-line treatment of advanced or recurrent EC or versus single-agent chemotherapy in advanced, recurrent or metastatic EC after one prior platinum-based chemotherapy.

## 1. Introduction

GLOBOCAN estimates of the worldwide incidence and mortality for 36 cancers in 185 countries reported 382,069 new cases of endometrial carcinoma (EC) and 89,929 deaths due to this cancer in 2018 [1]. EC has long been subdivided into two main categories termed type I endometrioid carcinoma and type II nonendometrioid carcinoma, but this dualistic model does not take into account the molecular, biological and pathological heterogeneity among each category [2,3]. On the basis of integrated genomic, transcriptomic and proteomic characterization of 373 ECs by array- and sequencing-based technologies, The Cancer Genome Atlas Research Network (TCGA) has proposed a novel classification into four categories termed polymerase ε (*POLE*) ultramutated, microsatellite instability (MSI) hypermutated, copy number low (endometrioid) and copy number high (serous-like) [4,5]. Talhouk et al. [6] have suggested a simplified, pragmatic, clinically applicable molecular-based classification system termed Proactive Molecular Risk Classifier for Endometrial Cancer (ProMisE) which does not require the expensive genomic methodology and that is applicable to formalin-fixed paraffin-embedded samples. This system based on mismatch repair (MMR) protein immunohistochemistry, POLE mutational analysis and p53 immunohistochemistry identifies four molecular subtypes that are similar but not identical to genomic TCGA subtypes. This classification has been validated in a large population-based cohort including 452 ECs from an independent institution [7]. In that study 28.1% of the tumors were MMR-deficient (MMR-d), 9.3% were POLE-mutant, 12.2% were p53-abnormal and 50.4% were p53-wild-type. ProMisE classification significantly correlated with progression-free survival (PFS) and overall survival (OS), with the most and the least favorable outcomes being the POLE-mutant subtype and the p53-abnormal subtype, respectively. The TransPORTEC consortium identified four very similar molecular subtypes using methods easily adopted in laboratories on formalin-fixed paraffin-embedded samples [8,9]. The analysis of 947 available early-stage endometrioid EC specimens from patients enrolled in the PORTEC-1 and PORTEC-2 trials, mostly at high-intermediate risk, revealed that 6% of the tumors were POLE-mutant, 9% were p53-mutant, 26% had MSI, and 59% had no specific molecular profile (NSMP) [9]. The prognosis was unfavorable in the p53-mutant subset, intermediate in the MSI and NSMP subsets and favorable in the POLE-mutant subset. The feasibility and usefulness of a diagnostic algorithm using immunostaining for p53 and MMR and mutation analysis of POLE have been confirmed in a study of tissue samples from 410 high-risk EC patients enrolled in the PORTEC-3 trial [10]. Twelve percent of the tumors were POLE-mutant, 33% were MMR-d, 32% had NSPM and 23% were p53-abnormal. The corresponding 5-year PFS rates were 98%, 72%, 74% and 48%, respectively (*p* < 0.001). Adjuvant combined chemotherapy and radiotherapy improved the 5-year PFS and 5-year OS compared with adjuvant radiotherapy alone in patients with p53-abnormal tumors (58.6% versus 36.2%, hazard ratio (HR) = 0.52, 95% confidence interval (CI) = 0.30–0.91; and, respectively, 64.9% versus 41.8%, HR = 0.55, 95% CI = 0.30–1.00). Conversely, the combined treatment failed to significantly improve the 5-year PFS and 5-year-OS compared with radiotherapy alone in patients with MMR-d EC (68.0% versus 75.5%, HR = 1.29, 95% CI = 0.68–2.45; and, respectively, 78.6% versus 84.0%) and in those with NSMP EC (79.7% versus 67.7%, HR = 0.68, 95% CI = 0.36–1.3; and, respectively, 89.3% versus 87.6%, HR = 0.68, 95% CI = 0.26–1.77). It is noteworthy that only one patient with a POLE-mutant EC (treated with radiotherapy alone) relapsed, resulting in a 5-year PFS and 5-year OS of 100% with combined chemotherapy and radiotherapy versus 96.6% with radiotherapy alone. Therefore, incorporation of the molecular classification into risk stratification systems is strongly suggested not only for prognostic purposes, but also to better plan adjuvant treatment as recommended by the recent European Society of Gynecological Oncology (ESGO)/European SocieTy for Radiotherapy & Oncology (ESTRO) and European Society of Pathology (ESP) guidelines [11].

At presentation, most EC patients are in an apparent early stage of disease and undergo primary surgery, usually via a mini-invasive approach, followed by observation or adjuvant treatment according to risk factors [11]. However, 11–19% of these patients will subsequently develop recurrent disease, often involving distant sites [12,13,14,15,16,17]. A paucity of patients with unresectable, locally advanced EC are treated with definitive radiotherapy or neoadjuvant chemotherapy followed by surgery or definitive radiotherapy [11,18].

Patients with metastatic or recurrent EC that is not suitable for surgery and/or radiotherapy are usually candidates for pharmacological treatments which are frequently associated with unsatisfactory clinical outcomes [19,20,21,22,23,24,25,26]. The aim of this review was to evaluate the results following treatment with chemotherapy, hormone therapy, molecularly targeted agents and immune checkpoint inhibitors and analyze the promising perspectives of research in this clinical setting. 

## 2. Chemotherapy

The combination of cisplatin (CDDP) + doxorubicin (DOX) has long been used in advanced or recurrent EC with objective response rates (ORRs) of 34–60%, median PFS of 5.3–8.0 months and median OS of 9.0–12.3 months, respectively, in chemo-naive patients [19,20,21]. The Gynecologic Oncology Group (GOG) 177 trial randomly assigned patients with advanced or recurrent disease to receive either DOX + CDDP + paclitaxel (PTX) every 3 weeks (Q3W) (TAP regimen) with granulocyte colony-stimulating factor support or DOX + CDDP Q3W [27]. The three-drug regimen produced higher ORRs (*p* < 0.01), longer PFS (median, *p* = 0.01) and longer OS (median, *p* = 0.037), but was associated with higher grade 2–3 peripheral neuropathy rates (39% versus 5%) [22]. In the GOG 209 randomized trial, the combination of PTX + carboplatin (CBDCA) AQ3W was not inferior to the TAP regimen in terms of PFS (HR = 1.032, 90% CI = 0.93–1.15) and OS (HR = 1.002, 90% CI = 0.9–1.12) [23]. Among the patients with RECIST 1.0 measurable disease, ORR for both arms was 52%. The combination of CBDCA + PTX is the standard first-line chemotherapy for advanced or recurrent EC [11,23,24,25,26].

McMeekin et al. [27] performed a pooled analysis of the data from 1203 patients with advanced or recurrent EC who received first-line single-agent or combination regimens, including DOX (12%), DOX + CDDP (63%), DOX + PTX (13%) or TAP (11%) within four randomized phase III GOG trials. ORRs were 42% in the whole series, 44% in patients with endometrioid carcinoma, 44% in those with serous carcinoma, 32% in those with clear cell carcinoma, 37% in those with mixed carcinomas and 39% in those with other histological types. In the logistic regression model, histology was not a predictive factor of response, but it was an independent prognostic variable of OS, with an HR of death of 1.2 (95% CI = 1.02–1.4) for serous carcinoma and of 1.51 (95% CI = 1.1–2.07) for clear cell carcinoma compared with all other histologic types. 

A multicenter retrospective cohort study assessed 262 patients with recurrent EC who underwent first-line platinum-based chemotherapy and who received second-line platinum-based chemotherapy at the time of relapse [28]. The ORRs for patients with a platinum-free interval (PFI) < 6 months, 6–11 months, 12–23 months and ≥ 24 months were 25%, 38%, 61% and 65%, respectively. Median PFS after second-line platinum-based chemotherapy was 4.4 months for patients with a PFI < 12 months versus 10.3 months for those with a PFI ≥ 12 months (*p* < 0.0001), and the corresponding median OS was 13.8 months versus 40.9 months (*p* < 0.0001). Therefore, the concept of platinum sensitivity is valid for recurrent EC, too. 

The chemotherapy options for patients not fit for platinum retreatment are very limited (Table 1). PTX produced ORR of 27–37% in patients who had received prior chemotherapy not including taxanes [29,30,31]. In the series of Lissoni et al. [29], this agent achieved a response in 22% of the nine platinum-resistant patients. Markman et al. [32] reported that weekly PTX (60–80 mg/m^2^) produced both objective responses and subjective improvement in three patients pretreated with CBDCA + PTX. 

The ORRs to the other single agents are unsatisfactory [29,30,31,32,33,34,35,36,37,38,39,40,41,42,43] (Table 1).

**Table 1:** Chemotherapy for metastatic, advanced or recurrent endometrial cancer.

**Platinum-Based Chemotherapy**					
**Study**	**Patient Profile**	**Treatment**	**ORR**	**Outcome (Months)** **Median PFS**	**Outcome (Months)** **Median OS**
Trope et al. (1984) [19]	19 ^a^	50 mg/m^2^ DOX + 50 mg/m^2^ CDDP q28	60%	NA	NA
van Wijk et al. (2003) [20]	90 ^a^	60 mg/m^2^ DOX + 50 mg/m^2^ CDDP q28	43%	8.0	9.0
Thigpen et al. (2004) [21]	131 ^a^	60 mg/m^2^ DOX + 50 mg/m^2^ CDDP q21	42%	5.7	9.0
Fleming et al. (2004) [22]	129 ^a^	60 mg/m^2^ DOX + 50 mg/m^2^ CDDP q21	34%	5.3	12.3
Fleming et al. (2004) [22]	134 ^a^	45 mg/m^2^ DOX + 50 mg/m^2^ CDDP d1 + 160 mg/m^2^ PTX d21	57%	8.3	15.3
Miller et al. (2020) [23]	656 ^a^	45 mg/m^2^ DOX + 50 mg/m^2^ CDDP d1 + 160 mg/m^2^ PTX d2 q21	52% ^^^	13.9	41.1
Miller et al. (2020) [23]	672 ^a^	CBDCA AUC 6 + 175 mg/m^2^ PTX q21	52% ^^^	13.2	37.0
Sovak et al. (2007) [24]	85 ^b^*	CBDCA AUC 5 + 175 mg/m^2^ PTX q21–28	43%	5.3	13.2
Pectasides et al. (2008) [25]	47 ^a^	CBDCA AUC 6+ PTX 175 mg/m^2^ q21	62%	15	25
Lorusso et al. (2019) [26]	108 ^a^	CBDCA AUC 5 + 175 mg/m^2^ PTX q21-	53%	10.5	29.7
**Non-Platinum-Based Chemotherapy**					
**Study**	**Patient Profile**	**Treatment**	**ORR**	**Outcome (Months)** **Median PFS**	**Outcome (Months)** **Median OS**
Sutton et al. (1994) [33]	40 ^b^	1.2 g/m^2^ IFO d1–4 q28	15%	DOR: 3.9 months	NA
Lissoni et al. (1996) [29]	19 ^c^	175 mg/m^2^ PTX q21	37%	NA	NA
Lincoln et al. (2003) [30]	44 ^c^	175 mg/m^2^ PTX q21	27%	DOR: 4.2 months	10.3
Homesley et al. (2008) [31]	15 ^c^	80 mg/m^2^ PTX q7	27%	DOR: 16.6 weeks	NA
Rose et al. (1996) [34]	22 ^b^	50 mg/m^2^ oral VP-16 q21	0%	NA	–
Muggia et al. (2004) [43]	42 ^b^	50 mg/m^2^ PLD q28	9.5%		8.2
Miller et al. (2002) [35]	22 ^b^	0.5–1.5 mg/m^2^ topotecan d1–5 q21	9%	DOR for CR: 6.9 months DOR for PR: 2.1 months	NA
Fracasso et al. (2004) [36]	52 ^b^	130 mg/m^2^ oxaliplatin q21	13%	DOR: 10.9 months	NA
Dizon et al. (2009) [37]	50 ^b^	40 mg/m^2^ ixabepilone q21	12%	2,9	8.7
McMeekin et al. (2007) [38]	248 ^b^	40 mg/m^2^ ixabepilone q21	15%	3.4	10.9
Miller et al. (2009) [39]	27 ^b^	900 mg/m^2^ pemetrexed q21	4%	2.7	9.4
Tait et al. (2011) [40]	23 ^b^	800 mg/m^2^ gemcitabine d1,8 q21	4%	1.7	NR
Makker et al. (2013) [41]		60 mg/m^2^ DOX q21	0%	2.1	5.8
Moreira et al. (2018) [42]		60 mg/m^2^ DOX q21	12%	4.4	8.1

^a^ No prior chemotherapy. ^b^ Prior chemotherapy. ^b^* Prior chemotherapy in 13 (15%) patients. ^c^ Prior chemotherapy not including PTX. ^^^ Among patients with RECIST 1.0 measurable disease. Legend: Pts, patients; RR, response rate; PFS, progression-free survival; OS, overall survival; DOX, doxorubicin; CDDP, cisplatin; NA, not available; PTX, paclitaxel; AUC, area under the curve; IFO, ifosfamide; DOR, duration of response; PTX, paclitaxel; VP-16, etoposide; PLD, pegylated liposomal doxorubicin; NR, not reached.

For example, DOX achieved ORR ranging from 0% to 12% in patients who progressed after PTX + CBDCA [41,42]. 

A phase III trial randomly allocated 496 patients with advanced, recurrent or metastatic EC who failed prior platinum-based chemotherapy to receive either ixabepilone or either PTX or DOX Q3W [38]. Patients previously treated with an anthracycline were randomized to either ixabepilone or PTX. The study was stopped early because the interim analysis of futility for OS favored the control arm (HR = 1.3, 95% CI = 1.0–1.7). 

## 3. Hormonal Therapy

Progestins, such as medroxyprogesterone acetate (MPA), megestrol acetate (MA) or hydroxyprogesterone caproate, produced objective responses in 11–25% of patients, with no significant advantage for any one agent [44,45,46,47] (Table 2). 

The highest chances of response were observed in patients with well-differentiated tumor grade and positive progesterone receptor (PR) status. In a GOG randomized trial, 1000 mg/daily MPA did not improve ORR or clinical outcome of patients compared with 200 mg/daily MPA [46]. 

Tamoxifen (TAM), which itself has a modest activity [48], could increase PR expression in cancer tissues and enhance the effectiveness of progestins in EC [49]. However, clinical studies on alternating treatment with TAM and progestins have given uncertain and unsatisfactory results [47,50,51]. For instance, in the study of Fiorica et al. [51], 80 mg MA twice daily (BID) for 3 weeks alternating with 20 mg TAM BID for 3 weeks achieved ORR of 31% in patients with extrapelvic disease compared to 14% in those with pelvic and/or vaginal disease. 

**Table 2:** Endocrine therapy for metastatic, advanced or recurrent endometrial cancer.

Study	Patient Profile	Treatment	ORR	Outcome (Months) Median PFS	Outcome (Months) Median OS
Piver (1980) [44]	114 ^a^	Different progestins (MAP, OH-P-caproate)	16%	NA	47.9 for OR
Podratz (1985) [45]	155	Different progestins (MAP, OH-P-caproate, medrogestone)	37%	NA	1 year: 40%; 2 years: 19%; 5 years: 8%
Thigpen (1999) [46]	145 ^a^	MPA, 200 mg	25%	3	11
	154 ^a^	MPA, 1000 mg	16%	2	7
Pandya (2001) [47]	20 ^a^	MA, 80 mg BID	20%	NA	12
	21 ^a^	80 mg MA + 10 mg TAM BID	19%	NA	9
Thigpen (2001) [48]	68 ^a^	TAM, 20 mg	10%	2	9
Whitney (2004) [50]	58 ^a^	MAP, 200 mg / TAM, 40 mg	33%	3	13
Fiorica (2004) [51]	56 ^a^	MA, 80 mg /TAM, 20 mg BID q21	27%	3	14
Rose (2000) [52]	23 ^b^	Anastrozole, 1 mg	9%	1	6
Ma (2004) [53]	28 ^b^	Letrozole, 2.5 mg	9%	NA	NA
Covens (2011) [54]	53 ^c^	Fulvestrant, 250 mg q28			
	22 RE−		0%	2	3
	31 RE+		16%	10	26
Emons (2013) [55]	35 ^d,^^	Fulvestrant, 250 mg q28	11%	2	13

^a^ No prior systemic therapy. ^b^ No more than one prior hormonal therapy and no prior chemotherapy. ^c^ Prior hormonal therapy and/or prior chemotherapy. ^d^ No prior hormonal therapy; prior chemotherapy in 14 pts; ^^^ RE+ and/or PR+. Legend: Pts, patients; RR, response rate; PFS, progression-free survival; OS, overall survival; MPA, medroxyprogesterone acetate; OH-P, 17α hydroxyprogesterone; medrogestone, 6,17α-dimethyl-6-dehydroprogesterone; MA, megestrol acetate; BID, bis in day.

Aromatase inhibitors and fulvestrant have limited efficacy [52,53,54,55].

Cyclin-dependent kinase 4/6 inhibitors have shown antineoplastic activity both in vitro against human EC cell lines and in vivo in xenograft models of EC [56,57]. In phase II trial NCT02657928, 55% of the 20 patients with recurrent estrogen receptor (ER)-positive EC were alive and progression-free at 12 weeks following treatment with ribociclib (400 mg orally daily) + letrozole (2.5 mg orally daily) [58]. Other trials are currently assessing the combination of cyclin-dependent kinase 4/6 inhibitors and hormonal agents in this clinical setting. The NCT02730429 study (ENGOT-EN3-NSGO/PALEO) is a randomized double-blind placebo-controlled phase II trial of palbociclib + letrozole versus placebo + letrozole in patients with ER-positive, advanced or recurrent EC [59]. NCT03643510 is a phase II study of fulvestrant + abemaciclib in hormone receptor-positive EC.

## 4. mTOR Inhibitors

The phosphatidylinositol 3-kinase (PI3K)/Akt/mammalian target of rapamycin (mTOR) pathway is often activated in EC, making it an attractive target for biological agents. However, ORRs to mTOR inhibitors are disappointing [60,61,62,63,64,65] (Table 3). 

In a phase II study, temsirolimus produced a response in 14% of chemo-naive patients and in 4% of those who had received prior chemotherapy [64]. Histological type, tumor grade, PTEN loss and the expression of molecular markers of the PI3K/Akt/mTOR pathway (mTOR, phosphorylated AKT (pAKT) and phosphorylated S6 (pS6)) did not correlate with the clinical outcome. In another study, ORRs to temsirolimus were similar in chemo-naive patients and in patients with prior chemotherapy [65].

Since the PI3K/AKT/mTOR pathway activation may lead to resistance to endocrine therapy, the combination of mTOR inhibitors and hormonal agents has been tested in EC. Letrozole + everolimus achieved an objective response in 31% and a clinical benefit in 40% of heavily pretreated patients [66]. No response was detected in patients with serous carcinoma, whereas patients with endometrioid histology and CTNNB1 mutations had the greatest clinical benefit from this treatment. The combination of metformin + letrozole + everolimus produced an objective response and a stable disease in 28% and 22% of the patients, respectively [67]. 

The NCT03008408 trial is a phase II randomized two-arm study of everolimus + letrozole with or without ribociclib in patients with advanced or recurrent EC. 

In the VICTORIA phase I–II study, patients with recurrent ER- and/or PR-positive advanced/metastatic EC were randomized (2:1) to either the target of rapamycin complex (mTORC) 1 and mTORC2 inhibitor vistusertib + anastrozole or single-agent anastrozole [68]. The combination arm showed a meaningful improvement in the 8-week PFS and median PFS compared with single-agent anastrozole (67% versus 39%, and 5.2 months versus 1.9 months, respectively) with acceptable toxicity. Fatigue, lymphopenia, hyperglycemia and diarrhea were the main vistusertib-related grade ≥ 2 adverse events (AE).

## 5. EGFR Inhibitors, HER-2 Inhibitors, MEK Inhibitors and MET Inhibitors

Gefitinib, erlotinib, lapatinib and trastuzumab have no meaningful activity [69,70,71,72] (Table 4). 

For example, erlotinib produced a response lasting 2–36 months in 12% of patients who had received one line of prior hormonal therapy and no chemotherapy [69]. Molecular analysis did not identify epidermal growth factor receptor (EGFR) mutations in responders. Trastuzumab produced no objective response in patients with HER2-positive EC after prior chemotherapy [71]. Conversely, in a phase II trial including 61 patients with HER2-positive serous carcinomas, the addition of trastuzumab (initial dose of 8 mg/kg IV, then 6 mg/kg) to CBDCA (area under the curve (AUC5)) + 175 mg/m^2^ PTX Q3W improved the median PFS in the whole series (12.6 versus 8.0 months, HR = 0.44; 90% CI = 0.26–0.76), in patients with stage III–IV disease (17.9 versus 9.3 months, HR = 0.40, 90% CI = 0.20–0.80) and in patients with recurrent disease (9.2 versus 6.0 months, HR = 0.14; 90% CI = 0.05–0.54) [73].

**Table 4:** EGFR inhibitors, Her-2 inhibitors, MEK inhibitor and MET inhibitor in metastatic, advanced or recurrent endometrial cancer.

Study	Patient Profile	Treatment	ORR	Outcome (Months) Median PFS	Outcome (Months) Median OS
Oza et al. (2008) [69]	32 ^a^	Erlotinib, 150 mg	12%	NA	NA
Leslie et al. (2013) [70]	26 ^b^	Gefitinib, 500 mg	4%	1.8	7.1
Leslie et al. (2012) [72]	30 ^b^	Lapatinib, 1500 mg	3%	1.8	7.3
Fleming et al. (2010) [71]	33 ^c,^^	4 mg/kg trastuzumab, then 2 mg/kg weekly	0%	1.81 *	6.8 *
				1.8 **	7.8 **
Ali-Ahmad et al. (2021) [74]	28 ^d^	8 mg/kg trastuzumab, then 6 mg/kg + 840 mg pertuzumab, then 420 mg	7%	28 weeks	NA
Coleman et al. (2015) [75]	52 ^b^	Selumetinib, 75 mg BID	6%	2	8
Dhani et al. (2020) [76]	E36 ^b^		14%	5	NR
	S34 ^b^		12%	4	NR
	UH32 ^b^	Cabozantinib, 60 mg	6%	3	NR

^^^ HER-amplified. * HER amplification by FISH/immunohistochemistry-positive. ** Immunohistochemistry-positive (HER2+, 3+). ^a^ Prior hormonal therapy; no chemotherapy. ^b^ Prior chemotherapy. ^c^ Prior chemotherapy regimens (total prior doxorubicin dose limited to 320 mg/m^2^). ^d^ ERBB2 amplification or overexpression (*n* = 22); ERBB2 mutations (*n* = 4); ERBB3 (*n* = 1); both ERBB2 amplification and mutation (No. 1). Legend: Pts, patients; RR, response rate; PFS, progression-free survival; OS, overall survival; NA, not available; E, endometroid; S, serous; UH, uncommon histology.

In the phase II basket TAPUR study, the combination of pertuzumab + trastuzumab Q3W obtained ORR of 7%, median PFS of 28 weeks in a cohort of EC patients with ERBB2 or ERBB3 amplified, overexpressing or mutated tumors [74]. One patient developed grade 3 muscle weakness, whereas there were no additional G3–4 AEs.

The MEK-1/2 inhibitor selumetinib achieved a response in 6% and 6-month PFS in 12% of heavily pretreated patients [75] (Table 4). The drug failed to meet the prespecified efficacy parameters for ORR (10%) and percentage of patients with 6-month PFS (15%). 

The MET inhibitor cabozantinib produced ORR of 14%, 12% and 6%, respectively, in patients with endometrioid, serous and uncommon histology [76]. A higher frequency of response was observed in patients with somatic CTNNB1 mutation and in those with concurrent somatic KRAS and PTEN or PIK3CA mutations (ORR of 40% and 25%, respectively).

## 6. Antiangiogenic Agents

The treatment of EC patients with multitarget tyrosine kinase inhibitors sorafenib, sunitinib, nintedanib and lenvatinib achieved disappointing results [77,78,79,80,81,82,83,84,85,86] (Table 5). 

For example, the single-agent lenvatinib targeting vascular endothelial growth factor (VEGF) receptor (VEGFR) 1–3, fibroblast growth factor receptor (FGFR) 1–4, platelet-derived growth factor receptor (PDGFR), RET and KIT produced ORR of 14% [81]. The most common AEs of any grade were fatigue/asthenia, hypertension, nausea/vomiting, decreased appetite and diarrhea. 

Trebananib is a peptide Fc fusion protein that binds angiopoetin-1 and -2, prevents their interaction with Tie2 receptors on endothelial cells and induces vascular remodeling antiangiogenetic activity in preclinical models of EC [83]. Trebananib obtained ORR of 3% and a 6-month PFS rate of 19% [84]. The most common toxicities of any grade were fatigue, anemia, constipation, hypertension, nausea, abdominal pain and limb edema.

**Table 5:** Antiangiogenic agents in metastatic, advanced or recurrent endometrial cancer.

Study	Patient Profiles	Treatment	ORR	Outcome (Months) Median PFS	Outcome (Months) Median OS
Nimeiri et al. (2010) [77]	Carcinoma 40 ^a^	Sorafenib, 400 mg BID	5%	3	11
	Carcinosarcoma 16 ^a^		0%	2	5
Castonguay et al. (2014) [78]	33 ^a^	Sunitinib, 50 mg	18%	3	19
Dizon et al. (2014) [79]	32 ^a^	Nintedanib, 200 mg BID	9%	3	10
Bender et al. (2015) [80]	48 ^a^	Cediranib, 30 mg	12%	4	12
Vergote et al. (2020) [81]	133 ^a^	Lenvatinib, 24 mg	14%	6	11
Moore et al. (2015) [84]	32 ^a^	Trebananib, 15 mg/kg	3%	2	7
Coleman et al. (2012) [85]	44 ^a^	Aflibercept, 4 mg/kg	7%	3	14
Aghajanian et al. (2011) [86]	52 ^a^	Bevacizumab, 15 mg/kg	13%	4	10

^a^ Prior chemotherapy. Legend: Pts, patients; RR, response rate; PFS, progression-free survival; OS, overall survival.

Aflibercept, a novel VEGF ligand-binding fusion protein that serves as a “decoy receptor” for VEGF, achieved ORR of 7% and a 6-month PFS rate of 41% [85] (Table 5). Grade > 3 AEs were cardiovascular, constitutional, hemorrhage, metabolic and pain. Two treatment-related deaths occurred due to gastrointestinal perforation and arterial rupture, respectively.

In a GOG phase II trial assessing single-agent BEV in recurrent or persistent EC, only 13% of patients experienced an objective response with a median response duration of 6 months [86]. The percentage of patients who had 6-month PFS was 35% for endometrioid carcinoma and 36% for serous carcinoma. Neither gastrointestinal perforation/fistula nor treatment-related death occurred.

The NRG Oncology/GOG-86P phase II trial randomly assigned 349 chemo-naive patients with stage III/IVA EC and measurable disease, stage IVB EC or recurrent EC to receive (i) CBDCA (AUC 5–6) + PTX (135–175 mg/m^2^) for six cycles with concurrent and maintenance BEV (15 mg/kg) Q3W or (ii) CBDCA (AUC 5) + PTX (135–175 mg/m^2^) for six cycles with concurrent temsirolimus (25 mg IV on days 1 and 8) and maintenance temsirolimus (25 mg IV on days 1 and 15) Q3W or (iii) ixabepilone (25–30 mg/m^2^) + CBDCA (AUC 5–6) with concurrent and maintenance BEV (15 mg/kg) Q3W [87]. The corresponding ORRs were 60%, 55% and 53%, respectively. PFS was not significantly increased in any experimental arm compared with historical controls represented by the patients of the CBDCA + PTX arm of the GOG 209 trial (23). Conversely, OS was improved in CBDCA + PTX + BEV arm (HR = 0.71, 92% CI = 0.55–0.91) but not in the other two arms, compared with historical controls. A subset analysis showed that combining chemotherapy with BEV could enhance both PFS and OS in patients with p53-mutant EC, which suggested that more aggressive tumors might draw a major benefit from angiogenesis inhibition [88]. 

In the MITO END-2 phase II randomized trial, the addition of concomitant and maintenance BEV (15 mg/kg) to CBDCA (AUC 5) + PTX (175 mg/m^2^) Q3W failed to significantly improve PFS (median, 13.7 versus 10.5 months, HR = 0.84, 95% CI = 0.5–1.3) and OS (median, 40.0 versus 29.7 months, HR = 0.71, 95% CI = 0.31–1.36) compared with chemotherapy alone in 108 patients with stage III–IV or recurrent EC [26].

## 7. Immune Checkpoint Inhibitors

Programmed death (PDO-1) inhibitors and PD-ligand (PD-L1) inhibitors are novel, very promising therapeutic tools [89,90,91,92,93,94,95,96,97,98] (Table 6).

The PD-1 inhibitor pembrolizumab (200 mg IV Q3W) produced ORR of 34% in 233 patients with previously treated, advanced, unresectable or metastatic MSI-H/MMR-d non-colorectal cancers of different types enrolled in the GOG 158 trial [89]. It is noteworthy that 70% of the 47 patients with EC experienced a ≥30% reduction in tumor size.

Pembrolizumab (10 mg/kg IV Q2W) resulted in an objective response in three (12%) and a stable disease in additional three (12%) of the 24 patients with heavily pretreated, advanced or metastatic PD-L1-positive EC included in the KEYNOTE-028 study [90]. The median duration of response was not yet reached, whereas the median duration of stable disease was 24.6 weeks. Twelve-month PFS and 12-month OS were 14.3% and 51.0%, respectively. All the three responders had endometrioid carcinoma, and one of them had a POLE mutation. This mutation was associated with high tumor mutational burden (TMB) and elevated expression of several immune checkpoint genes [91].

The cumulative analysis of 149 patients with MSI-H/MMR-d cancers of 15 different types enrolled in five clinical trials found that pembrolizumab produced ORR of 40%, with 78% of responses lasting ≥ 6 months [92]. 

The PD-L1 inhibitor avelumab (10 mg/kg IV Q2W) produced an objective response in 27% of patients with MMR-d EC. PD-L1 expression, number of tumor-infiltrating lymphocytes and TMB did not correlate with the response [93]. Three of the tumors not responding to avelumab harbored mutations of Janus kinase 1 or beta-2 microglobulin, which have been associated with acquired resistance to immune checkpoint inhibitors in melanoma [94]. Conversely, avelumab had no meaningful activity in MMR-proficient (MMR-p) ECs. 

In the Garnet trial, the anti-PD1 dostarlimab (500 mg IV Q3W for four doses, then 1000 mg Q6W) resulted in a complete response in nine and a partial response in 21 of the 71 patients with MMR-d EC, with ORR of 42% ranging from 40% in type I EC to 48% in type II EC [95]. The median duration of response was not reached after a median follow-up of 11.2 months, and 77% of patients were alive at 12 months. The most common G3 AEs were anemia, colitis and diarrhea. 

The anti PD-L1 durvalumab (1500 mg IV Q4W) produced ORR of 47% in women with MMR-d EC progressing after 0–3 lines of chemotherapy and 3% in those with MMR-p EC progressing after 1–3 chemotherapy regimens [97]. AEs occurred in 20% of the patients and mainly consisted of G1–2 hypothyroidism, hyperthyroidism, pneumonitis and hypoadrenalism. Only one case of G3 hepatitis was reported. 

The analysis of the subprotocol National Cancer Institute Molecular Analysis for Therapy Choice (NCI-MATCH) trial showed that the anti PD-1 nivolumab was associated with a 12-month PFS of 46% and a median OS of 17.3 months in 42 highly pretreated patients with MMR-d non-colorectal cancer of different types [98]. Nivolumab produced an objective response in 46% of 13 EC patients. The most common G1 AEs were fatigue, anemia, rash and hypoalbuminemia.

The addition of cabozantinib to nivolumab improved ORR (25% versus 17%) and median PFS (5.3 versus 1.9 months) compared with single-agent nivolumab, with an increased toxicity mainly represented by G1–2 diarrhea, liver enzymes elevation, fatigue and hypertension [99].

In the open-label single-arm phase Ib/II study 111/KEYNOTE-146, the combination of lenvatinib (20 mg daily) + pembrolizumab (200 mg Q3W) achieved a 24-week response rate of 38% in 108 patients with highly pretreated EC, ranging from 64% in patients with MSI-H/MMR-d to 36% in those with microsatellite stable (MSS)/MMR-p tumors [100]. There was a robust depth of response, with 84% of patients having decreased tumor lesions from baseline and 30% having a maximum decrease of ≥50%. A response was detected in 36% and 40% of patients with PD-L1-positive and PD-L1-negative tumors, respectively, but these data must be considered with caution because of the uncertainty of the cutoff for PD-L1 positivity. With the exception of hypothyroidism, which occurred in 44% of the patients, the safety profile of the combination was comparable to that of each agent. Lenvatinib could modulate tumor immunity by decreasing the suppressive tumor-associated macrophage population [101]. A post hoc analysis found that selected AEs, such hypertension, fatigue, nausea/vomiting, diarrhea, decreased appetite/weight loss, hypothyroidism, palmar–plantar erythrodysesthesia, musculoskeletal pain, stomatitis and proteinuria, developed within the first 10 weeks of treatment. G ≥ 3 fatigue, hypertension and nausea occurred in ≥5% of patients [102]. AEs may be managed with concomitant supportive medications and lenvatinib dose modifications.

How et al. [103], who assessed pembrolizumab + lenvatinib in 70 patients with recurrent EC in a “real-world” clinical setting, found that a reduced starting dose of lenvatinib of 14 mg was associated with a better toxicity profile and similar ORR, PFS and OS compared to the standard 20 mg dose.

## 8. Conclusions

The clinical outcome of patients with metastatic or recurrent EC not suitable for surgery and/or radiotherapy is very poor, and the pharmacologic treatment options have a mainly palliative intent. The combination of PTX + CBDCA is the standard chemotherapy, able to achieve ORRs of 43–62%, a median PFS of 5.3–15 months, and a median OS of 13.2–37.0 months respectively, whereas hormonal therapy is sometimes used in selected patients with slow growing, ER- and/or PR- positive EC.

Whereas single-agent aromatase inhibitors or m-TOR inhibitors have limited activity, the combination of these agents has resulted in an objective response in approximately 30% of patients who failed after prior chemotherapy regimens. The association of cyclin-dependent kinase 4/6 inhibitors and hormonal agents is currently under evaluation.

Clinical trials of EGFR inhibitors, multi-tyrosine kinase inhibitors, and HER2 inhibitors have usually resulted in very disappointing results. However, a phase II trial of patients with advanced or recurrent HER2 positive serous carcinomas showed that the combination of trastuzumab + CBDCA + PTX significantly improved PFS compared with chemotherapy alone. The addition of BEV to CBDCA + PTX improved OS in the NRG Oncology/GOG-86P trial, but not in the MITO END-2 trial. These conflicting results might be due to both the different chemotherapy settings (first-line versus second-line chemotherapy) and the heterogeneous baseline patient characteristics

We must take into consideration that the low number of patients included in many of the clinical trials might have impacted either or both the outcome of the trials or the potential reproducibility of trial results.

In recent years the implementation of biological knowledge of EC, which has led to a molecular-based classification system, and the positive results obtained with immune checkpoint inhibitors have offered novel and promising tools for the treatment of patients with metastatic or recurrent disease. Anti- PD-1 and anti-PD-L1 antibodies have achieved ORRs ranging from 27% to 47% in highly pretreated patients with MMR-d EC.

Pembrolizumab received accelerated US Food and Drug Administration (FDA) approval for patients with unresectable or metastatic MSI-H/MMR-d solid tumors regardless of tissue of origin, that have progressed after prior treatment and who have no alternative treatment options.

In the KEYNOTE-146 study the combination of lenvatinib + pembrolizumab has produced an objective response in 36% of patients with MSS/MMR-p EC. Based on this study, this combination was granted accelerated approval for the patients with advanced, not MSI-H or MMR-d EC who have progressed after systemic therapy and who are not candidates for curative surgery or radiotherapy.

Several randomized phase III trials with immune check point inhibitors are currently ongoing in EC (Table 7).

For instance, four trials are investigating the addition of immune checkpoint inhibitors to PTX + CBDCA in primary advanced or recurrent EC (NCT03603184, NCT03914612, NCT04269200, NCT03981796A), one trial (NCT03884101) is comparing pembrolizumab + lenvatinib versus CBDCA + PTX as first-line treatment of advanced or recurrent EC, and another trial (NCT03517449) is comparing pembrolizumab + lenvatinib versus single- agent chemotherapy in patients with advanced, recurrent or metastatic EC after one prior platinum-based chemotherapy.

## Figures and Tables

**Table 3:** mTOR inhibitors for metastatic, advanced or recurrent endometrial cancer.

Study	Patient Profile	Treatment	Pts	ORR	Outcome (Months) Median PFS	Outcome (Months) Median OS
Ray-Coquard et al. (2010) [60]	44 ^a^	Everolimus, 10 mg	44 ^a^	4%	3	8
Slomovitz et al. (2015) [66]	35 ^a^	2.5 mg letrozole + 10 mg everolimus	35 ^a^	31%	3	14
Soliman et al. (2015) [67]	54 ^a^	500 mg metformin + 2.5 mg letrozole + 10 mg everolimus BID	54 ^a^	28%	6	20
Oza et al. (2011) [64]	29 ^b^	Temsirolimus, 25 mg weekly	29 ^b^	14%	7	NA
	25 ^a^	Temsirolimus, 25 mg weekly	25 ^a^	4%	3	NA
Fleming et al. (2014) [65]	11 ^c^	25 mg temsirolimus weekly + 80 mg TAM BID / 20 mg TAM BID for 3 weeks	11 ^c^	9%	5 ^^^	11^^^
	10 ^d^		10 ^d^	20%	8 ^^^	21^^^
	21		21	14%		
	29 ^c^	Temsirolimus, 25 mg weekly	29 ^c^	24%		
	21 ^d^		21 ^d^	19%		
	50		50	11%		
Colombo et al. (2013) [61]	45 ^a^	Ridaforolimus, 12.5 mg d1–5 q15	45 ^a^	11%	6-PFS: 18%	NA
Oza et al. (2015) [63]	48 ^c^	Ridaforolimus, 40 mg d1–5/week	48 ^c^	0%	6	10
	47 ^c^	Comparator *	47 ^c^	4%	2	10
Tsoref et al. (2014) [62]	34 ^e^	Ridaforolimus, 40 mg q1–5 q15	34 ^e^	9%	NA	NA
	11 ^a^		11 ^a^	9%		
	23 ^b^		23 ^b^	9%		
Heudel et al. (2021) [68]	49 ^a,§^	125 mg vistusertib BID + 1 mg anastrozole	49 ^a,§^	24%	PFS-8w: 67%	PFS: 5
	24 ^a,§^	Anastrozole, 1 mg	24 ^a,§^	17%	PFS-8w: 39%	2

^^^ Both arms combined. * MPA, MA, CBDCA, PTX, topotecan, DOX or PLD. ^§^ ER/PR positive. ^a^ Prior chemotherapy. ^b^ No prior chemotherapy; prior hormonal therapy. ^c^ Prior chemotherapy, no hormonal therapy. ^d^ No prior chemotherapy, no hormonal therapy. ^e^ Prior hormonal therapy; prior chemotherapy only permitted on adjuvant setting. Legend: Pts, patients; RR, response rate; PFS, progression-free survival; OS, overall survival; MA, megestrol acetate; TAM, tamoxifen; MPA, MPA, medroxyprogesterone acetate; CBDCA, carboplatin; PTX, paclitaxel; PLD, pegylated liposomal doxorubicin.

**Table 6:** Immune checkpoint inhibitors in metastatic, advanced or recurrent endometrial cancer.

Study	Patient Profile	Treatment	ORR	Outcome (Months) Median PFS	Outcome (Months) Median OS
Ott et al. (2017) [90]	24 ^a^ PD-L1+	Pembrolizumab, 10 mg/kg	12%	2	NR
Kostantinoupoulos et al. (2019) [93]	16 ^a^ MMRp	Avelumab, 10 mg/kg	6%	2	7
	15 ^a^ MMRd		27%	4	NR
Oaknin et al. (2020) [95]	71 ^a^ MMRd	Dostarlimab, 500 mg	42%	8	NR
Liu et al. (2019) [96]	15 ^a,b^ PD-L1+	Atezolizumab, 1200 mg q21	13%	1	10
Antillet al. (2021) [97]	35 ^a^ MMRd	Durvalumab, 1500 mg	47%	8	NR
	36 ^a^ MMRp		3%	2	12
Azad et al. (2020) [98]	13 ^a^ MMRd	Nivolumab, 3 mg/kg	46%	NA	NA
Lheureux et al. (2020) [99]	18	Nivolumab, 240 mg	17%	2	NA
	36 ^a^	240 mg nivolumab + 40 mg cabozantinib	25%	5	NA
Makke et al. (2020) [100]	94 ^a^ MSS/MMRp	20 mg lenvatinib + 200 mg pembrolizumab	36%	4	NR
	11^a^ MSH-I/MMRd		64%	19	

^a^ Stratification for MSI status; ^b^ MSS (No. 12), MSI-H (No. 1), MSI status unknown (No. 2). Legend: Pts, patients; RR, response rate; PFS, progression-free survival; OS, overall survival; PD-L1, programmed death ligand; MSS, microsatellite stability; NR, not reached; MMRp, mismatch repair-proficient; MSI-H, microsatellite instability-high; MMRd, mismatch repair-deficient.

**Table 7:** Ongoing clinical trials with immune checkpoint inhibitors in metastatic, advanced or recurrent endometrial cancer.

Trial	Details
NCT03603184 AtTEnd (Atezolizumab Trial in Endometrial Cancer)	Phase III double-blind randomized placebo-controlled trial of atezolizumab in combination with PTX and CBDCA in patients with newly diagnosed EC with residual disease after surgery either measurable or evaluable, with inoperable stage III–IV EC naïve to first-line systemic treatment and with recurrent EC not yet treated for relapsed disease (estimated study completion: December 2023)
NCT03914612 NRG-GY018)	Phase III randomized placebo-controlled study of pembrolizumab in addition to PTX and CBDCA for measurable stage III or IVA, stage IVB or recurrent EC (estimated study completion: June 2023)
NCT04269200 DUO-E/GOG-3041/ENGOT-EN10	Randomized multicenter double-blind placebo-controlled phase III study of first-line CBDCA + PTX in combination with durvalumab followed by maintenance durvalumab with or without olaparib in patients with newly diagnosed advanced or recurrent EC (estimated study completion: March 2025)
NCT03884101 (ENGOT-en9/MK-7902-001, LEAP-001)	Phase III randomized open-label study of pembrolizumab plus lenvatinib versus chemotherapy (CBDCA + PTX) for first-line treatment of advanced or recurrent EOC (estimated study completion: April 2023)
NCT03517449 (KEYNOTE-775)	Phase III randomized open-label phase III study comparing the efficacy and safety of pembrolizumab plus lenvatinib versus treatment of physician’s choice (weekly PTX or PLD) in patients with advanced, recurrent or metastatic EC (estimated study completion: November 2023)
NCT04262089 (PAM)	Neo-adjuvant Pembrolizumab in dMMR/ POLE-EDM Uterine Cancer Patients: a Feasibility Study
NCT04486352 (EndoMAP)	A Phase IB/II Multi-Cohort Study of Targeted Agents With Atezolizumab for Patients With Recurrent or Persistent Endometrial Cancer
NCT04106414 (CA017-056)	A Phase II Trial of IDO-Inhibitor, BMS-986205, and PD-1 Inhibitor, Nivolumab, in Patients With Recurrent or Persistent Endometrial Cancer or Endometrial Carcinosarcomas (CA017-056)
NCT04463771 (POD1UM-204)	An Umbrella Study of INCMGA00012 Alone and in Combination With Other Therapies in Participants With Advanced or Metastatic Endometrial Cancer Who Have Progressed on or After Platinum-Based Chemotherapy (POD1UM-204)
